# The Significance of Appendiceal Diverticulosis to the Emergency General Surgeon: A Case Report and Literature Review

**DOI:** 10.7759/cureus.70582

**Published:** 2024-09-30

**Authors:** Kesav Aditya Vijayagopal, Natasha Khan, Richard C Newton

**Affiliations:** 1 General Surgery, University Hospital Sussex National Health Service (NHS) Foundation Trust, Chichester, GBR; 2 Surgery, University Hospital Sussex National Health Service (NHS) Foundation Trust, Chichester, GBR

**Keywords:** acute complicated appendicitis, appendiceal diverticula, appendix tumors, complex appendix surgery, tumor appendix

## Abstract

Acute appendicitis is a common acute general surgical presentation; however, variants in the appendicular anatomy and the presence of cancers can complicate the surgical management of this condition. Appendiceal diverticula (AD) are rare pathological outpouchings along the appendix body. These outpouchings complicate and obscure the surgical planes, making adequate surgical resection of the appendix difficult. We report here the surgical management of a rare case of AD with a benign neuroma that highlights the importance of adequate pre-operative planning and variations in the standard anatomy. Through the literature review, the association of AD with cancers arising from the appendix is also presented.

## Introduction

Appendicitis is a common general surgical presentation, with an incidence of 81 cases per 100,000 persons a year in the UK [1]. Appendiceal diverticula (AD) are pathological mucosal herniations through the muscular wall of the appendiceal body and macroscopically present as outpouchings on the appendix [2,3]. AD can be classified into congenital and acquired forms and is evident in 0.004%-2.1% of appendicectomy specimens. Autopsy studies describe a prevalence between 0.2% and 0.66% [3,4]. We report the operative management of an unusual case of AD with a benign neuroma that highlights the importance of pre-operative planning in variations of standard anatomy. Through a review of the available literature on AD, we identify the clinical significance and importance of AD in a wider clinical context.

## Case presentation

A 48-year-old Caucasian male presented to the Accident and Emergency Department with a two-day history of migratory right iliac fossa abdominal pain with raised inflammatory markers, white cell count of 13.4, neutrophilia of 10.6, and CRP of 26; all other standard blood tests were normal (Table 1). A CT scan was subsequently performed to confirm the diagnosis. This revealed an appendix dilated to 14 mm diameter with peri-appendicular fat stranding, thickening of the peritoneal reflections, and enlarged ileocolic lymph nodes.

**Table 1:** Patient blood test results with the reference ranges eGFR, estimated glomerular filtration rate

Laboratory values	Values	Reference ranges
WBC count	13.4 × 10^9^/L	4.3-10.3 × 10^9 ^/L
Neutrophils	10.6 × 10^9 ^/L	1.5-8 × 10^9 ^/L
Lymphocytes	1.7 × 10^9 ^/L	1.0-4.0 × 10^9 ^/L
Monocytes	1 × 10^9 ^/L	0.2-1 × 10^9 ^/L
Eosinophils	0.1 × 10^9 ^/L	0-0.5 × 10^9 ^/L
Sodium	139 mmol/L	135-145 mmol/L
Potassium	4 mmol/L	3.5-5.3 mmol/L
Chloride	106 mmol/L	95-108 mmol/L
Urea	4.5 mmol/L	2.5-7.8 mmol/L
Creatinine	91 µmol/L	45-84 µmol/L
eGFR	86 mL/minute/1.73 m^2^	>90 mL/minute/1.73 m^2^
Albumin	44 g/L	30- 45g/L
CRP	26 mg/L	<5 mg/L

**Figure 1:**
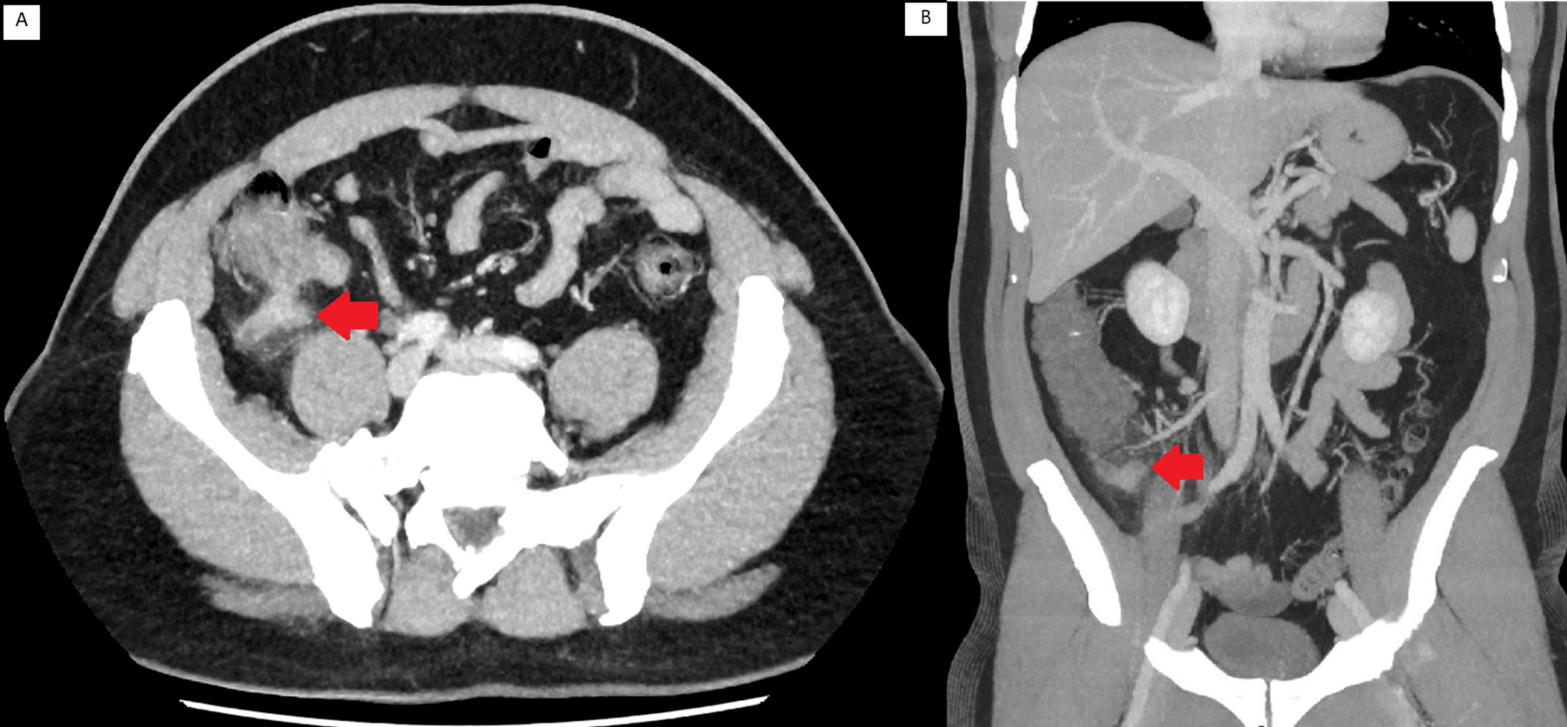
CT images of appendicitis with appendiceal diverticula The above are images taken from the pre-operative CT scan. Appendiceal diverticula are noted and indicated by the red arrows. Given the inflammatory fat stranding and clear margins, this was initially perceived to be a contained perforation and or abscess, which had formed from the appendix.

Careful examination of the base of the appendix on CT images below led to suspicions of perforation and possible abscess formation toward the base of the appendix. Intra-operatively, the tip of the appendix was readily identified, and dissection was commenced from the tip of the appendix down to what was presumed to be the base (Figure 1).

**Figure 2:**
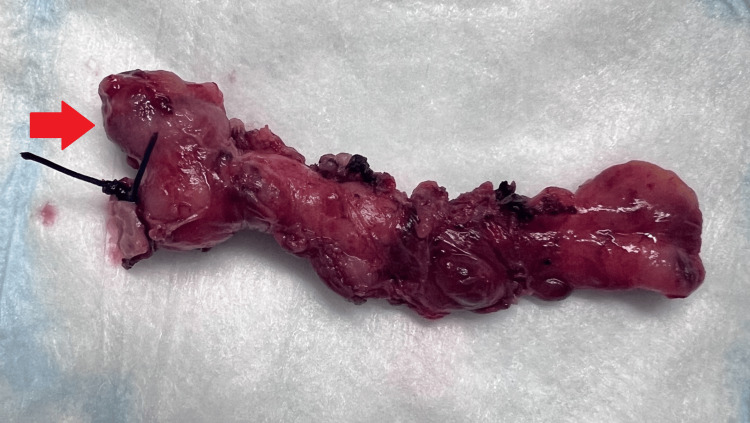
Gross pathological specimen of the appendix containing appendiceal diverticula Gross specimen of the appendix with the base of the appendix is indicated by the presence of a surgical stitch to the left of the image. The red arrow indicates the large appendiceal diverticula arising just distal to the base of the appendix.

With inflammatory adhesions of the pre-ileal fat pad obscuring the operative plane, an AD of the appendicular body was initially perceived as the cecal pole. Further careful dissection was required to operatively define the AD along its margins and identify the true cecal pole. The gross pathologic specimen is seen in Figure 2.

**Figure 3:**
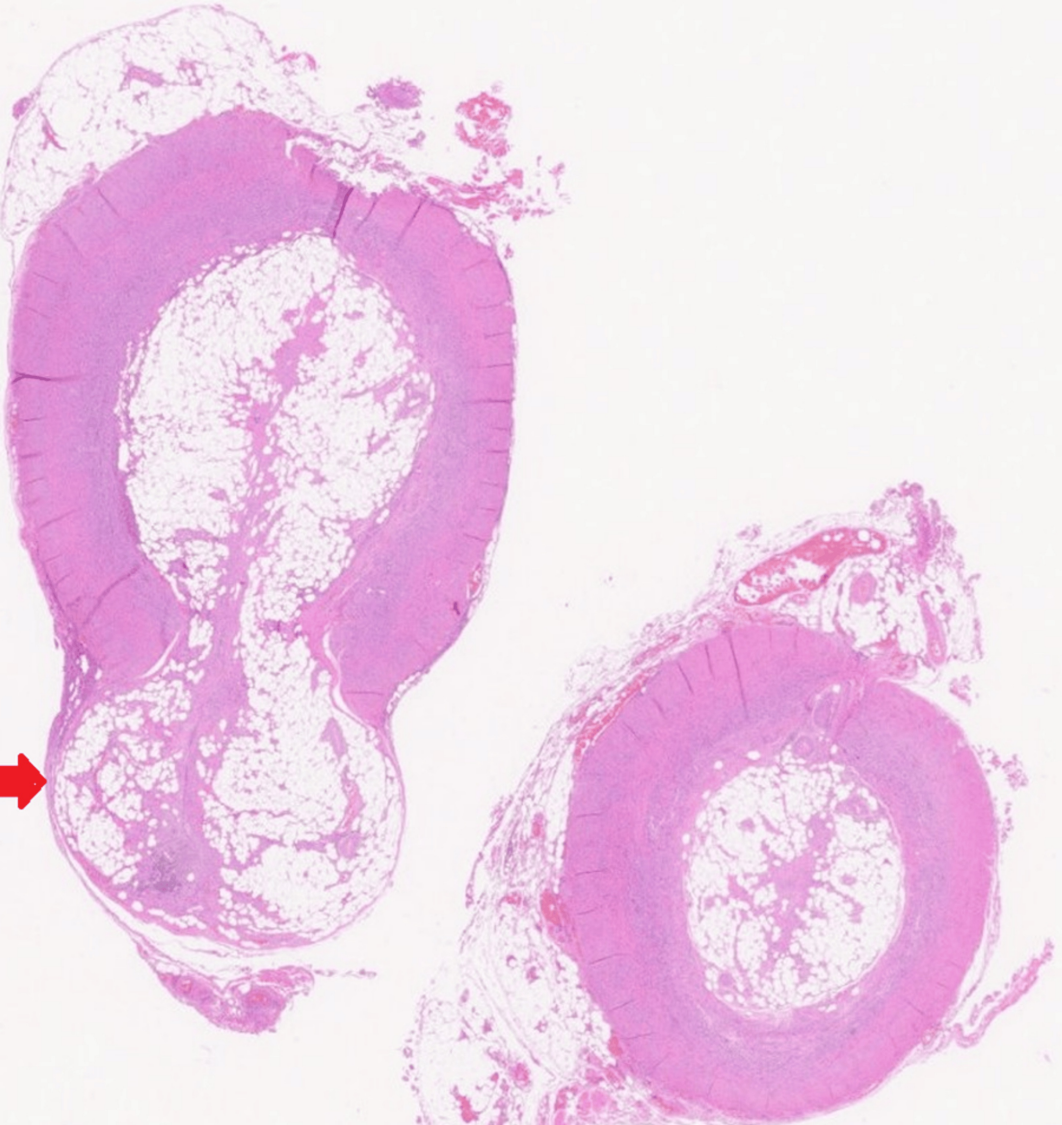
Histology of the appendix and appendiceal diverticula The red arrow indicates the thin-walled diverticulum arising from the body of the appendix. Within the diverticulum, the presence of a neuroma was noted. The section on the right of the image shows a segment more distally, with thickening of the appendiceal wall and inflammatory infiltration in keeping with a diagnosis of acute appendicitis.

Microscopically, as seen in Figure 3, there was a benign neuroma (also called a fibrous obliteration) of the appendiceal lumen with spindle cell proliferation replacing the lumen of the appendix. A thickened appendiceal wall was also noted in keeping with acute appendicitis. Pathology reports confirmed that this was a blind-ending outpouching of appendiceal tissue containing fecolith.

## Discussion

Although there is no clear pathogenesis for AD, we hypothesize that, in our case, a fecolith trapped within the lumen of the appendix resulted in a chronic low-grade inflammatory process causing raised intraluminal pressures within a blind-ending pouch [3]. This chronic inflammatory process also led to hypertrophy of neuroendocrine cells, resulting in the formation of a neuroma as identified in histology [5]. These two factors likely caused a weakness within the muscular layers of the appendix, resulting in the formation of an AD [3,5].

There are, at present, no established treatment guidelines for AD. A review of available literature noted an association between AD and appendicular neoplasms. Lamp et al. have identified that in 42% of cases of low-grade appendiceal mucinous neoplasms (LAMNs), there was an associated AD. Dupré et al. reported that 48% of AD had an associated concomitant appendiceal neoplasm [4,6]. In another large-volume study of 4,413 appendicectomy specimens, of the identified 39 cases of appendiceal diverticulosis, 17 (43.6%) were noted to have an appendiceal neoplasm and or a neoplastic precursor. This is in comparison to the 1.2% for the non-diverticular appendix [7]. This has led to the recurring theme within recent literature that the presence of appendiceal diverticulosis is a putative marker of local/regional neoplasms [2,4,7]. The American Joint Committee on Cancers (AJCC) Eighth Edition gives guidance on the management of appendicular neoplasms (summarized in Table 2, split into the four major subtypes); however, there is a lack of clear guidelines for the management of AD, which, as we have seen, acts as a putative marker for an underlying cancer [8].

**Table 2:** Summary of the various appendiceal neoplastic conditions and their management according to AJCC Eighth Edition guidelines *Cytoreductive surgery and HIPEC are recommended for patients with peritoneal dissemination. **Systemic chemotherapy has been shown to be effective in improving overall survival in patients with AJCC stage IV G2 or G3 mucinous adenocarcinoma [9]. ***There is a paucity of literature describing the role of an oncological right hemicolectomy, with several analyses published showing limited benefits, although this practice is still recommended by both the North American and European Neuroendocrine Tumor societies [9]. AJCC, American Joint Committee on Cancers; HAMN, high-grade appendiceal mucinous neoplasm; HIPEC, hyperthermic intraperitoneal chemotherapy; LAMN, low-grade appendiceal mucinous neoplasm

Type	Subtypes	Treatment
Colonic type	Adenocarcinoma	As colonic adenocarcinoma
Mucinous	Serrated polyp with or without dysplasia	Appendicectomy alone
	LAMN	Appendicectomy ± HIPEC*
	HAMN	Appendicectomy + oncological right hemicolectomy ± cytoreductive surgery vs. HIPEC
	Mucinous adenocarcinoma with or without signet cells	Appendicectomy + oncological right hemicolectomy ± systemic chemotherapy** ± cytoreductive surgery** vs. HIPEC**
Goblet cells	Typical goblet cell carcinoma	Appendicectomy ± oncological right hemicolectomy*** ± cytoreductive surgery** vs. HIPEC**
	Signet ring cell type	Appendicectomy ± oncological right hemicolectomy*** ± cytoreductive surgery** vs. HIPEC**
	Poorly differentiated cell type	Appendicectomy ± oncological right hemicolectomy*** ± cytoreductive surgery** vs. HIPEC**
Neuroendocrine	-	Appendicectomy ± right hemicolectomy

Given its significance in appendiceal neoplasms, Ng et al. have proposed that in the presence of an AD, surgeons must perform en-bloc removal of the meso-appendix for adequate oncological margins [2]. In the event an AD is identified incidentally, some authors argue for a prophylactic appendicectomy, given the high risk of neoplasms and perforation [4,6,7].

Table 3 below shows a summary of the identified literature on the association of AD as a marker of neoplasm or neoplastic precursors.

**Table 3:** Summary of the literature on the association between appendicular neoplasms and appendiceal diverticula AD, appendiceal diverticula

Authors	Type of publication	Total number of cases in the cohort	Number of patients with AD	Description
Dupre et al., 2008 [4]	Retrospective cohort study of appendicectomy specimens	1,361	23	48% of AD cases had associated neoplasms (carcinoid and mucinous neoplasms)
Lamps et al., 2000 [6]	Retrospective cohort study of mucocele/adenoma/diverticulum of appendicectomy	32	8	42% of low-grade mucinous neoplasms seen in association with AD
Kallenbach et al [7]	Retrospective cohort study of appendicectomy specimens	4,413	39	43.6% of AD with neoplasm/precursor neoplasm vs. 1.2% in non-AD samples
Lipton et al., 1989 [10]	Retrospective cohort study of appendicectomy specimens	3,342	56	4× increase in risk of perforation in those with AD
AbdullGaffar, 2009 [11]	Retrospective cohort study of appendicectomy specimens	1,234	4	Increased rate of perforation (30×), hemorrhage requiring blood products, and abscess formation
Osada et al., 2012 [12]	Retrospective cohort study of appendicectomy specimens	156	7	Increased incidence of perforation ranging from 30% to 66%

## Conclusions

Following our case, the importance of recognizing this rare variant of appendicular anatomy has clearly been demonstrated. There are various learning points we would like to highlight from this case. At the very least, careful operative planning and study of pre-operative images under an experienced clinician is required for appropriate and timely management of AD. In our case, the operative plane was obscured by the presence of a pre-ileal fat pad covering the cecal pole and base of the appendix. This could easily have resulted in transection through the body of the AD, leaving a large appendicular stump and risking a feculent leak and future stump appendicitis. Secondly, the available literature demonstrates an association of AD with an underlying neoplasm of the appendix. Given the potential role AD plays in appendicular neoplasms, further research is required to elucidate this association, which will form the basis of future treatment guidelines.
